# Lipid-Targeted Atherosclerotic Risk Reduction in Older Adults: A Review

**DOI:** 10.3390/geriatrics7020038

**Published:** 2022-03-25

**Authors:** Lauren J. Hassen, Steven R. Scarfone, Michael Wesley Milks

**Affiliations:** Department of Internal Medicine, The Ohio State University College of Medicine, Columbus, OH 43210, USA; lauren.hassen@osumc.edu (L.J.H.); steven.scarfone@osumc.edu (S.R.S.)

**Keywords:** lipid-lowering therapy, statin, hyperlipidemia, low density lipoprotein

## Abstract

Aggressive lipid-lowering lifestyle modifications and pharmacologic therapies are the cornerstones of the primary and secondary prevention of atherosclerotic cardiovascular disease events. While statins are highly effective, inexpensive, and generally well-tolerated medications, many clinicians and patients express uncertainty regarding the necessity of statin treatment in older adults. Citing concerns such as polypharmacy, muscle symptoms, and even potential cognitive changes with statins, many patients and health care providers elect to de-intensify or discontinue statin therapy during the process of aging. A lack of clear representation of older individuals in many clinical trials and practice guidelines may contribute to the ambiguity. However, the recently prevailing data and practice patterns supporting the benefits, safety, and tolerability of a variety of lipid-lowering therapeutics in older adults are discussed here, with particular mention of a potential protective effect from incident dementia among a statin-treated geriatric population and an admonishment of the historical concept of “too-low” low-density lipoprotein cholesterol (LDL-C) levels.

## 1. Introduction

**Figure 1:**
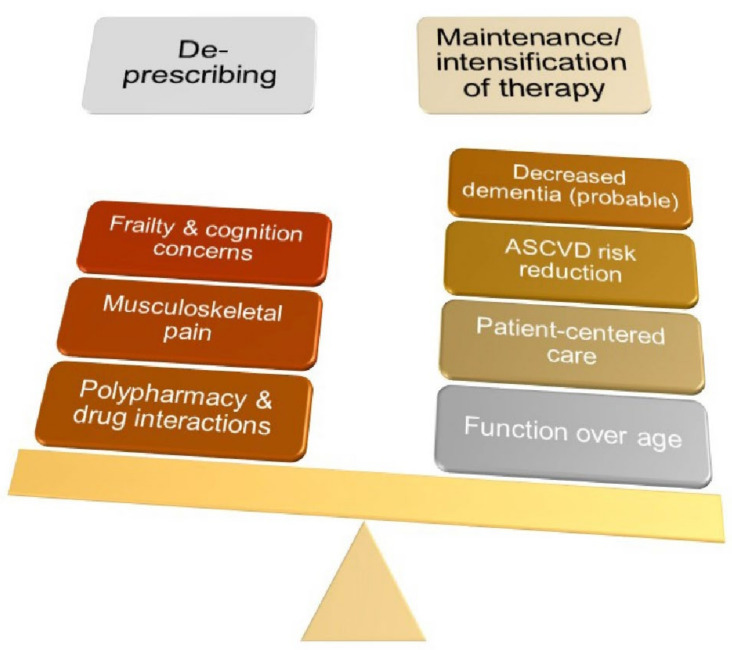
Conceptual diagram. Various potential issues influencing the risk and benefit balance in favor of or against the use of lipid-lowering pharmacotherapies in older individuals are listed.

As has been widely reported, the US population is aging. Currently, about 15% of the population is aged 65 years or older [1]. By 2050, this proportion is projected to increase to 22%, representing about 85 million older people. In this population of older adults, as in the general population, cardiovascular disease is the leading cause of death [2]. While there are an abundance of data addressing atherosclerotic cardiovascular disease (ASCVD) risk reduction in the general population, there are a number of considerations unique to older adults (Figure 1). This review will highlight those considerations and is intended to serve as a reference for any clinician caring for older adults.

## 2. Definitions

This review utilizes the same definition of ASCVD as in the 2018 American Heart Association/American College of Cardiology (AHA/ACC) guidelines on the management of blood cholesterol [3]. Specifically, ASCVD is defined to encompass stable angina, unstable angina, myocardial infarction (MI), ischemic stroke, transient ischemic attack, peripheral arterial disease (PAD) including aortic aneurysm, or any arterial revascularization, all of atherosclerotic origin [3]. References to “older adults” are meant to include those aged 65 or older. However, many of the current ASCVD risk reduction guidelines are defined to include adults up to age 75. Therefore, clinicians may find this review most useful when caring for those adults age >75, the care of whom is poorly directed by current guidelines. The primary prevention of ASCVD refers to treatment prior to the development of ASCVD, to preclude or delay disease onset. The secondary prevention of ASCVD refers to intervening upon clinically recognized ASCVD, to inhibit or delay the progression of disease.

## 3. Summary of ASCVD Risk Reduction Interventions

**Figure 2:**
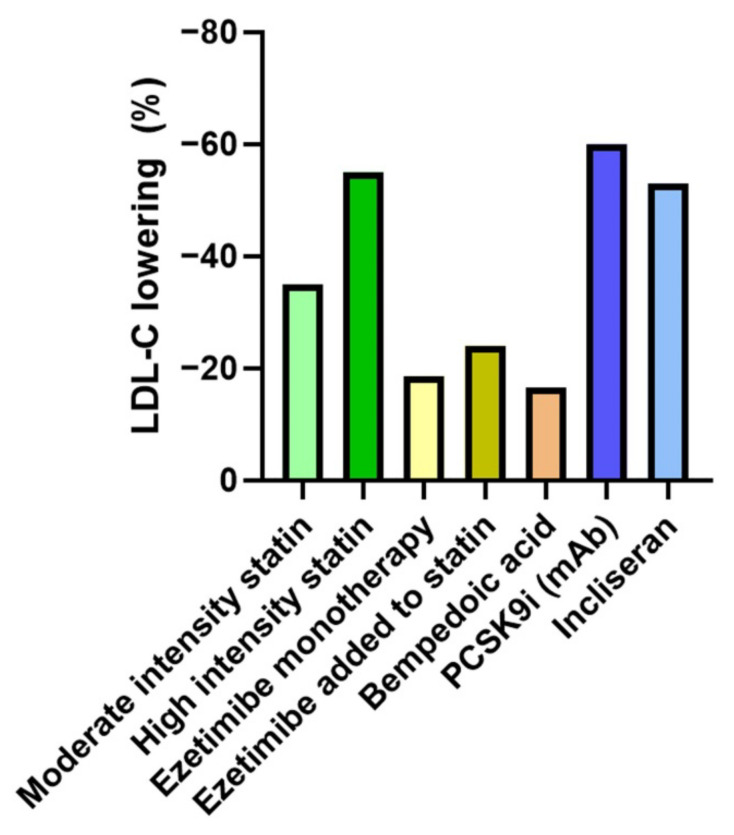
Approximate LDL-C reduction with selected lipid-lowering drugs. Approximate and/or average LDL-C reductions with selected agents are shown. High intensity statins include atorvastatin 40 to 80 mg/day and rosuvastatin 20 to 40 mg/day [3]; percentage LDL-C reductions are abstracted from the results of the Statin Therapies for Elevated Lipid Levels compared Across doses to Rosuvastatin (STELLAR) trial [4], with depicted examples of achieved LDL-C reduction using rosuvastatin 40 mg/day (high intensity statin) and simvastatin 20 mg (moderate intensity statin). The effects of ezetimibe monotherapy (cited in Mach et al. [5]) and the additional LDL-C reduction when combined with statin therapy in the IMPROVE-IT trial [6] are shown. Bempedoic acid therapy is discussed later in the work. The effects of PCSK9 inhibitor therapies including monoclonal antibody (mAb) agents (evolocumab and alirocumab) [5] and the small interfering RNA (siRNA) agent incliseran [7] are depicted.

The mainstays of ASCVD risk reduction are therapeutic lifestyle modifications and lipid-lowering medications, namely HMG-CoA reductase inhibitors (statins), cholesterol absorption inhibitors (ezetimibe), proprotein convertase subtilisin/kexin type 9 (PCSK9) inhibitors (evolocumab, alirocumab), and icosapent ethyl. More recent additions to the pharmacotherapeutic armamentarium for LDL-C reduction include bempedoic acid, incliseran, and evinacumab. For most patients, significant reductions in atherogenic cholesterol, as well as in ASCVD risk, are achievable via these means (Figure 2).

Other lipid-lowering agents such as bile acid sequestrants, niacin, and fibrates are available but have a narrower evidence base to support treatment-related ASCVD risk reduction. Antiplatelet therapy in primary ASCVD prevention is beyond the scope of this review, while briefly noting that the U.S. Preventive Services Task Force recently suggested a recommendation against (Grade D) low-dose aspirin for primary prevention in adults aged 60 years or older, which is consistent with the 2019 ACC/AHA guidance to avoid the routine use of low-dose aspirin for primary ASCVD prevention [8]. Additionally, diligent and patient-centered treatment of hypertension in older adults is a critically important topic, particularly given the associated risk of dementia [9], which is also beyond the scope of this work.

### 3.1. Lifestyle Modification

The foundation of ASCVD risk reduction strategies is an adherence to a heart-healthy lifestyle, both relating and not relating to the mechanism of atherogenic cholesterol reduction. Heart-healthy lifestyle patterns generally include changes in diet composition, optimization of quantity and/or intensity of physical activity, tobacco cessation, and the maintenance of a healthy body weight. These interventions should serve as first-line treatments for most patients in both primary and secondary ASCVD prevention, because of their low associated risks, as well as the health benefits extending beyond the cardiovascular system. The strongest available data support tobacco use cessation. A recent meta-analysis of over 500,000 patients showed a cardiovascular mortality hazard ratio of 2.07 for active smokers and 1.37 for former smokers when compared to never smokers, with the risk increasing with cigarette consumption in a dose-response manner and decreasing with time since cessation for former smokers [10]. Similarly, there are strong nonrandomized data showing an association between physical activity and a reduced incidence of cardiovascular disease and mortality, again in a dose-response relationship [11,12,13].

The association between dietary modification and ASCVD outcomes is exceptionally difficult to study, in part due to the vast heterogeneity in dietary patterns, inconsistent adherence to and persistence of interventions, and the long lag time between changes and the outcomes of interest. Thus, few randomized data are available, but it appears that a diet high in fruits, vegetables, nuts, legumes, whole grains, and fish (commonly referred to as a Mediterranean diet) is effective for primary prevention of ASCVD [8,14]. Additional challenges relate to the often-unclear degree to which the health benefits of dietary changes are mediated through lipoprotein level modifications, such as LDL-C reduction, or other benefits. For example, saturated fat reduction tends to result in LDL-C lowering, but for many individuals, carbohydrate restriction can lead to reductions in blood sugar and body weight, which can result in benefits relating to the attenuation of the detrimental effects of metabolic syndrome and/or type 2 diabetes mellitus [15].

### 3.2. HMG-CoA Reductase Inhibitors (Statins)

Statins are the most well-studied of the medication classes discussed here and constitute the backbone of pharmaceutical approaches to ASCVD risk reduction. They have proven effective for secondary [16,17,18,19,20,21], as well as primary, prevention of ASCVD, [21,22,23,24,25] and notably carry a mortality benefit in most of the cited trials. The studies cited are predominantly large, randomized trials involving diverse populations. Statins are associated with fairly minimal risks and costs, which typically are not treatment-limiting, despite what can often be a maligned reputation among those concerned about adverse drug effects. However, there are multiple statin-associated side effects which bear mentioning. Myalgias are perhaps the most common effects perceived as related to statin therapy, affecting anywhere from 1 to 10% of patients taking the medication in randomized trials [3], although the rate may be even higher based on real-world experience. Of course, distinguishing between drug-associated and unrelated muscle complaints can be highly challenging in clinical practice, noting that among National Health and Nutrition Examination Survey respondents, 22% of statin users and 17% of non-statin users reported muscle pain [26]. A recent n-of-1 trial suggests that as much as 90% of the perceived muscle symptom burden associated with atorvastatin 20 mg daily may also be reported with placebo [27].

Whether muscular complaints occur with greater frequency among statin-treated older vs. younger individuals is a critical yet challenging issue to study, noting that muscular pain, weakness, and sarcopenia are more frequent in general among older individuals. The STOMP study recruited healthy, previously statin-untreated subjects who underwent physical activity and muscle symptom quantification, as well as strength testing, before and after treatment with atorvastatin 80 mg vs. a placebo [28]. Participants of 55 years or older (vs. <40 years, without further advanced age stratification performed) treated with atorvastatin did have reduced activity counts; however, no significant changes in muscle strength, endurance, or aerobic performance were observed. Interestingly, a recent study of older (aged 64 ± 4 years) symptomatic (n = 16) and asymptomatic (n = 16) statin users demonstrated improvements in muscle performance and capillarization determined on biopsy among both groups after a 12-week endurance and resistance exercise training program [29]. Symptomatic statin users in the study reported an improved quality-of-life following the training program, which provides an encouraging signal that the presence of muscle pain or weakness with the initiation of statin therapy does not necessarily imply permanent statin treatment limitation or an irreversibility of symptoms with the continuation of statin treatment [29]. Finally, one meta-analysis of statin trials including individuals aged ≥65 years found no increased risk of myopathy in statin-treated older adults [30]. Rarely, muscular side effects are more serious, ranging from myositis to rhabdomyolysis or autoimmune myopathy [3].

Other potential statin-associated adverse effects include new-onset diabetes mellitus (DM), elevated transaminase levels, and possibly cognitive changes [3]. The excess risk of DM with statin treatment was examined in a large (n = 91,140) meta-analysis of 13 trials, with the finding that statin therapy vs. placebo was associated with a 9% increased risk for incident DM, which is relatively modest and arguably far outweighed by the benefits of statin treatment in ASCVD risk reduction [31]. While the excess risk for DM was slightly higher in trials that included older participants [31], it is notable that age is a risk factor for the development of type 2 DM, so some would argue that older adults with prediabetes and/or at risk for type 2 DM may have diabetes onset only slightly accelerated by statin therapy. Cognitive issues will be discussed in detail in this review, paying particular attention to their impact on the population of older adults. Statins are predominantly available in oral, once-daily, generic formulations, and so have minimal financial and pill burden. However, a notable consideration in statin choice is drug-drug interactions. Many drugs, including cyclosporine, clarithromycin, protease inhibitors, and others, interact significantly with statins and require careful attention when prescribing [32].

### 3.3. Cholesterol Absorption Inhibitors (Ezetimibe)

The cholesterol absorption inhibitor ezetimibe is another available pharmaceutical intervention for ASCVD risk reduction. The IMProved Reduction of Outcomes: Vytorin Efficacy International Trial (IMPROVE-IT) showed that, when added to simvastatin 40 mg daily for the secondary prevention of ASCVD, ezetimibe provides further risk reduction [33]. With regards to primary prevention, the 2018 AHA/ACC guidelines suggest that ezetimibe may be reasonable in intermediate-risk adults who would be recommended to take a high-intensity statin but can only tolerate a moderate-intensity statin [3]. In the open-label, randomized and blinded Ezetimibe Lipid-Lowering Trial on Prevention of Atherosclerosis in 75 or Older (EWTOPIA 75) study of adults aged ≥75 years across 363 centers in Japan, patients randomized to ezetimibe vs. usual care had marked reductions in the primary composite outcome (sudden cardiac death, MI, coronary revascularization, or stroke) over a median follow up of 4.1 years [34].

Regarding risks, a recent review showed that ezetimibe did not increase any adverse outcomes when added to statin therapy, though the evidence quality was low [35]. Ezetimibe is available as an oral, once-daily, generic formulation, so it has few pill burden considerations, though it remains more expensive than statin therapy. Significant drug-drug interactions involving ezetimibe are limited, such as with cholestyramine or cyclosporine [36].

### 3.4. PCSK9 Inhibitors (Alirocumab, Evolocumab, Incliseran)

Proprotein convertase subtilisin/kexin 9 (PCSK9) inhibitors, including alirocumab (Praluent^®^) and evolocumab (Repatha^®^), function by increasing the LDL-C receptor concentration by inhibiting degradation, thus decreasing serum LDL-C levels by approximately 60%. They have been an option for LDL-C and ASCVD risk reduction since 2015. These medications were studied in the Further Cardiovascular Outcomes Research With PCSK9 Inhibition in Subjects With Elevated Risk (FOURIER) and Evaluation of Cardiovascular Outcomes After an Acute Coronary Syndrome (ODYSSEY OUTCOMES) trials, which showed that the PCSK9 inhibitors, when added to statin therapy for secondary prevention, further reduced the recurrence of ASCVD events [37,38]. While an oral PCSK9 inhibitor is under development [39], current agents are available only as injection formulations. Importantly, PCSK9 inhibitors are well-tolerated; adverse responses are limited only to injection-site reactions, and notably there is no association with myalgias [29]. PCSK9 inhibitors are expensive and have received scrutiny for uncertain cost-effectiveness [40], the estimates for which can vary greatly between primary and secondary prevention populations [41]. PCSK9 inhibitors have not yet been found to interact with any other medications [42], but patients who have difficulty with or unwillingness for self-injection every two weeks would not be good candidates for evolocumab or alirocumab treatment.

Incliseran (Leqvio^®^) is a novel agent consisting of a synthetic small interfering RNA that is specifically taken up by hepatocytes and inhibits PCSK9 transcription. Dosing includes a single subcutaneous injection, again at three months, followed by every six months thereafter, with an achieved mean LDL-C reduction of 53% at day 180 post initiation in trials leading to approval [7,43]. Given that the drug is intended for administration only by health care professionals, this may prove to be an ideal option for ASCVD-affected patients with limited statin tolerance and trepidation or inability for self-injection; in addition, coverage by medical rather than drug benefits could result in a lower cost exposure to patients, which may be a particular concern among many older Medicare beneficiaries. Among the 1833 participants in the clinical studies leading to approval, 54% were aged 65 years or older, without apparent differences in effectiveness or safety among older vs. younger people [7].

Given the dramatic LDL-C reduction that can be achieved with PCSK9 inhibitors, many clinicians have wondered whether there exists a “too-low” LDL-C concentration for optimal health. The Evaluating PCSK9 Binding Antibody Influence on Cognitive Health in High Cardiovascular Risk Subjects (EBBINGHAUS) subgroup consisted of 1974 FOURIER trial participants with a mean age 62.7 years, among whom investigators found no difference in cognitive function assessments or subjective self-assessments of cognition, even among those with LDL-C < 25 mg/dL [44]. A subsequent expanded analysis of FOURIER participants (n = 22,655) also found no difference in the incidence of patient-reported cognitive decline among evolocumab-treated patients vs. placebo, including those with ultra-low (<20 mg/dL) LDL-C [45].

### 3.5. Bempedoic Acid

Bempedoic acid is a novel oral small molecule agent used to promote LDL-C reduction that functions as an ATP citrate lyase (ACL) inhibitor, thus reducing cholesterol biosynthesis upstream of HMG-CoA reductase [46]. As a prodrug, bempedoic acid requires modification by an enzyme present almost solely in the liver; thus, in concept, it avoids active drug penetrance into muscular tissue and averts potential adverse muscle symptoms. As a stand-alone drug (Nexletol^®^), bempedoic acid has been demonstrated in the Cholesterol Lowering via Bempedoic Acid, an ACL-Inhibiting Regimen (CLEAR) Harmony trial to lower LDL-C by 16.5%, although it is also sold in combination with ezetimibe (Nexlezet^®^). The mean age of the participants was 66.1 years, and muscular outcomes did not differ between the treated vs. placebo groups, although notably gout and tendon rupture events were higher among aggregated clinical trial participants as cited by the prescribing instructions [47]. Given that bempedoic acid is also indicated for patients with familial hypercholesterolemia or established ASCVD [47], this agent might be seen as most helpful in those who are unable to receive subcutaneous injections, given the much lower degree of LDL-C reduction attainable with bempedoic acid as compared with PCSK9 inhibitors.

### 3.6. Icosapent Ethyl

Another medication for ASCVD risk reduction functioning outside of the LDL-C lowering pathway is icosapent ethyl (Vascepa^®^). Icosapent ethyl is a highly purified omega-3 fatty acid preparation containing only eicosapentaenoic acid (EPA) without docosahexaenoic acid (DHA). It is approved for use in patients with established cardiovascular disease or the presence of diabetes with other risk factors for cardiovascular disease, who have moderate hypertriglyceridemia (i.e., >150 mg/dL) despite statin therapy. This approval is based on the results of the REDuction of Cardiovascular Events with Icosapent Ethyl-Intervention Trial (REDUCE-IT), which showed that in this patient population, icosapent ethyl significantly reduces the risk of ischemic events [48]. Of note, there was no significant increase in the fishy eructation effect, which can be a frequent cause for the discontinuation of other fish oil preparations. Cost is a concern, as icosapent ethyl is typically more expensive than other ASCVD risk reduction options, aside from the PCSK9 inhibitors, although a generic formulation has recently become available. The medication is dosed orally as two capsules taken twice daily, which poses a pill burden consideration. No major drug-drug interactions have been identified, but interestingly there was an increased incidence of hospitalization for atrial fibrillation or flutter in icosapent ethyl-treated patients (3.1% vs. 2.1% with placebo) [48], which is worthy of consideration particularly noting that the incidence of atrial arrhythmias increases markedly with advancing age.

While some historical concerns regarding omega-3 polyunsaturated fatty acid products and bleeding exist, there was no difference in the risk of serious adverse bleeding events in the REDUCE-IT trial [48], and, in fact, the randomized Omega-3 Fatty Acids for Prevention of Postoperative Atrial Fibrillation (OPERA) trial of adults undergoing cardiac surgery (mean age 64 years) suggested that perioperative fish oil supplementation is associated with a lower risk of perioperative bleeding complications [49]. Of note, the strongly beneficial ASCVD risk reduction effect observed in REDUCE-IT has not been replicated in trials of some other mixed (EPA and DHA) omega-3 polyunsaturated fatty acid products (e.g., of omega-3 carboxylic acids in the negative Long-Term Outcomes Study to Assess Statin Residual Risk Reduction with EpaNova in High Cardiovascular Risk Patients With Hypertriglyceridemia [STRENGTH] trial) [50]. Prior precedent for the benefit of EPA-only products (e.g., the Japan EPA Lipid Intervention Study [JELIS] trial) [51] highlights the need for continued study regarding the specific omega-3 compound, dose, and placebo used in trials intended to determine the maximal potential benefits of omega-3 treatments. For example, some have suspected an adverse effect of the mineral oil placebo in REDUCE-IT.

### 3.7. ANGPTL3 Inhibitors

Evinacumab (Evkeeza^®^) is a human monoclonal antibody that belongs to the novel therapeutic class of angiopoietin-like 3 (ANGPTL3) inhibitors; given that ANGPTL3 itself is an inhibitor of lipoprotein lipase and endothelial lipase, the suppression of ANGPTL3 activity can drive a marked reduction in triglyceride levels [52]. However, as informed by a study of individuals with loss-of-function variants [53], ANGPTL3 inhibition also results in a remarkable decrease in LDL-C, independent of the LDL receptor. As such, evinacumab has been initially labeled for patients with homozygous familial hypercholesterolemia [54], which is a highly rare disorder (approximately 1 in 300,000) that classically results from homozygous or compound heterozygous pathogenic variants in LDLR, APOB, or PCSK9 leading to minimal intact LDL receptor function [52]. Evinacumab has also been shown to lower LDL-C by 50% or more at the maximum dose in less selective populations with refractory hypercholesterolemia, i.e., LDL-C > 70 mg/dl with ASCVD, or >100 mg/dl without ASCVD [55]. Notably, in the safety trials leading to approval, the mean age of treated patients was 48 years, with the oldest treated individual 75 years old [54]; thus, much future study remains necessary to evaluate this agent among older adults in need of ASCVD risk reduction. As a dual-target therapy that lowers both LDL-C and triglyceride levels, evinacumab is also under ongoing study to evaluate a potential impact in reducing the risk of recurrent pancreatitis in the setting of severe hypertriglyceridemia (clinical trials identifier NCT04863014).

## 4. Secondary Prevention of ASCVD in Older Adults

While the focus of this review is on treatment strategies for the primary prevention of ASCVD in older adults, a brief review of secondary prevention strategies will be included as well. In short, the 2018 AHA/ACC guidelines provide recommendations for patients with ASCVD who are at a very high risk of developing future ASCVD events which are separate from recommendations for other patients with ASCVD. Patients are considered very high-risk if they have either a history of multiple major ASCVD events (acute coronary syndrome within the past year, MI/stroke ever, symptomatic PAD) or one major ASCVD event with multiple risk-enhancing factors (age >65, heterozygous familial hypercholesterolemia, a history of coronary artery bypass graft or percutaneous coronary intervention, heart failure, diabetes, hypertension, chronic kidney disease, active smoking, LDL-C >100). For very high-risk patients, regardless of age, the recommended strategy for secondary prevention is the pursuit of an LDL-C <70 and non-HDL <100 mg/dL, to be achieved first with maximally tolerated statin, then by adding ezetimibe, and finally by the addition of a PCSK9 inhibitor. In contrast, for patients with ASCVD who are not considered to be at a very high risk for future events, the guidelines differ based on patient age. In patients up to age 75, it is a class I recommendation to start a high intensity statin. In patients older than 75, it is a class IIa recommendation to start a moderate or high intensity statin or to continue a high intensity statin if it is well tolerated, after a clinician-patient discussion of the risks, benefits, and costs associated with statin therapy, and a consideration of patient frailty [3].

## 5. Primary Prevention of ASCVD in Older Adults

In contrast to the considerable quantity of data and the guidelines available to assist clinicians with the secondary prevention of ASCVD in older patients, fewer resources exist to navigate primary prevention, as patients >75 years of age are often excluded from or poorly represented in clinical studies, particularly those with high quality prospective data.

The 2018 AHA/ACC guidelines do include several class IIb recommendations for primary prevention in older adults. First, it may be reasonable to initiate a moderate intensity statin in adults 75 years or older with an LDL-C of 70–189 mg/dl. Second, in adults 75 years of age or older, it may be reasonable to stop statin therapy when functional decline, multimorbidity, frailty, or reduced life expectancy limit the potential benefits [3].

The guidelines cite evidence from several randomized controlled trials (RCT) and meta-analyses that support the use of statins in the elderly, while acknowledging that data for patients >80 years of age are sparse. Glynn et al. [56] performed a secondary analysis of 5695 participants >70 years of age in the Justification for Use of Statins in Prevention: An Intervention Trial Evaluating Rosuvastatin (JUPITER) trial [25], including participants without hyperlipidemia (LDL-C <130 mg/dL) but with elevated high-sensitivity C-reactive protein >2.0 mg/L. Participants who were randomized to rosuvastatin 20 mg daily had a significant reduction in the frequency of major adverse cardiovascular events (MACE) compared with the placebo group, although there was no significant decrease in all-cause mortality. The Heart Outcomes Prevention Evaluation 3 (HOPE-3) trial randomized participants at intermediate risk of ASCVD to rosuvastatin 10 mg daily or a placebo [57]; a meta-analysis of the data from the JUPITER [25] trial combined with 3086 participants >70 years of age from the HOPE-3 [57] trial showed a similar reduction in MACE [58]. A particularly large meta-analysis of 27 trials by Mihaylova et al. in 2012 showed a 17% relative risk reduction in major vascular events per 1 mmol/L reduction in LDL-C through statin use in participants >70 years of age [59]. Similar meta-analyses of eight statin RCTs performed in 2013 by Savarese et al. [60] and in 2015 by Teng et al. [61] showed significant reductions in varying types of composite and individual MACE in participants >65 years of age, but neither identified any mortality benefit [60,61]. Interestingly, in the Physicians Health Study, Orkaby et al. found a significant reduction in all-cause mortality, though not in MACE, in 7213 male physicians >70 years of age who reported statin use on annual questionnaires [62,63].

In contrast, several RCTs are also mentioned in the 2018 ACC/AHA guidelines which do not support the use of statins in the elderly for primary prevention. Subgroup analyses for participants without previous vascular disease in the Pravastatin in Elderly Individuals at Risk of Vascular Disease (PROSPER) trial did not show a benefit of statin use [64]. In a post hoc secondary analysis of the Antihypertensive and Lipid-Lowering Treatment to Prevent Heart Attack Trial–Lipid-Lowering Trial (ALLHAT-LLT), no reductions in all-cause mortality or MACE were found in participants >65 years of age [65]. Importantly, both studies utilized pravastatin 40 mg daily rather than higher intensity agents [64,65]. These negative trial results provide a counterpoint to the aforementioned studies, and the inconsistency of the available data likely reduced the strength of the guideline recommendations to Class IIb, in addition to concerns about adverse drug effects in this age group, as outlined below.

Since the 2018 ACC/AHA guidelines were published, two notable retrospective cohort studies examining primary prevention in the elderly have been added to the literature. Orkaby et al. studied new statin initiation in over 57,000 veterans aged >75 years who were free of ASCVD, for a mean follow up period of seven years. They identified significant reductions in all-cause mortality, cardiovascular mortality, and MACE [66]. Mortensen and Nordestgaard analyzed a large cohort of individuals without preexisting ASCVD, diabetes, or statin use at baseline, including nearly 14,000 individuals aged 70–100 years. They found a significant increase in the odds of developing an MI and/or ASCVD with every 1 mmol/L increase in LDL-C, with a trend towards a larger effect size with age. They estimated that the number needed to treat (NNT) with a moderate intensity statin to prevent one atherosclerotic event in five years was 42 for individuals aged 80–100 years, and 88 for individuals aged 70–79 years, vastly lower than the NNT for younger age groups [67]. Of course, age is one of the most impactful risk factors for ASCVD, so with increasing age comes a higher event risk, and thus a greater potential for absolute risk reduction with treatment. While retrospective in nature, these studies that include large numbers of older adults are important additions to the literature, supporting and adding to the conclusions of earlier prospective studies with smaller enrollment.

In addition to the cited primary prevention data, several pertinent meta-analyses mixing primary and secondary prevention patients have been published since the 2018 ACC/AHA guidelines were released. The Cholesterol Treatment Trialists’ Collaboration analyzed 28 RCTs and observed a reduction in major vascular events per 1 mmol/L reduction in LDL-C in all age groups through the initiation or intensification of statin therapy; however, there was a trend to a smaller effect size with increasing age that was not statistically significant [68]. A subgroup analysis of primary prevention participants was not performed. Gencer et al. examined 29 RCTs utilizing both statin and non-statin therapies, including ezetimibe and PCSK9 inhibitors, in participants aged at least 75 years. The investigators identified a 26% reduction in the risk of major vascular events per 1 mmol/L reduction in LDL-C. This effect was not significantly different when statin and non-statin therapies were analyzed separately, and the benefit of LDL-C lowering persisted for each component of the primary endpoint (cardiovascular death, MI, stroke, and coronary revascularization) [69]. These important studies underscore the benefits of continuing to pursue a lower LDL-C in older patients, either with statins or other therapies, though neither focuses on a primary prevention cohort specifically.

## 6. Primary Prevention in Older Adults with Diabetes Mellitus

Appropriately, diabetes mellitus (DM) has long been considered a significant risk factor for ASCVD in all adults. For most adults with DM, the 2018 ACC/AHA guidelines recommend initiating at least a moderate intensity statin without the need for further risk assessment. In adults with DM who have multiple ASCVD risk factors, it is a class IIa recommendation to prescribe high-intensity statin therapy with the aim to reduce LDL-C levels by 50% or more [3]. However, the guidelines do make special mention of adults older than 75 years of age with DM, as they are considered to be at a particularly high risk [70,71]. First, as a class IIa recommendation, in adults older than 75 years of age with DM who are already on statin therapy, it is reasonable to continue statin therapy. Second, as a class IIb recommendation, in adults older than 75 years of age with DM, it may be reasonable to initiate statin therapy after a clinician-patient discussion of the potential benefits and risks [3]. Notably, data in this area are lacking, as some of the already limited literature regarding primary prevention in older adults excludes patients with preexisting DM [56,67].

## 7. ASCVD Risk Assessment in Older Adults

For adults between the ages of 20 and 79 years, ASCVD risk can be easily estimated using the pooled cohort equations (PCEs); however, adults older than 79 years of age are excluded from the PCEs. Additionally, if PCEs are used, most older adults will meet an indication for statin therapy based on age alone. Notably, age is strongly weighted in the equations, and critics of the PCEs will note that there may be many healthy older adults, as well as younger individuals with poorly incorporated risk factors (e.g., family history of premature ASCVD, metabolic syndrome, hyperlipoprotein(a), etc.), that will be misclassified by this method. Thus, the PCE calculation approach does not allow for a nuanced risk assessment of older adults, for whom an individualized consideration of comorbid conditions, frailty, and reduced life expectancy is paramount. Uncertainty in risk assessment often leads to fewer prescriptions for statin therapy in adults >75 years of age, likely causing some to be deprived of potentially marked benefits [72].

To assist with this, the 2018 ACC/AHA guidelines provide a class IIb recommendation: for those 76–80 years of age, it may be reasonable to measure coronary artery calcium (CAC) in order to exclude those patients with a CAC score of 0 from statin therapy. Many studies have examined the power of CAC for risk stratification, but few include data for adults >75 years of age. Mortensen et al. studied the use of CAC scoring and carotid plaque burden (cPB) to clarify the 2013 ACC/AHA guideline indications for statin therapy in the BioImage Study [73,74]. They prospectively studied 5805 individuals aged 55 to 80 years without ASCVD for a median follow up of 2.7 years. Participants with CAC or cPB scores of 0 (32% and 23% of the study participants, respectively) were excluded from statin therapy, and participants with CAC score >100 were considered to be clearly statin eligible. This reclassification from the 2013 ACC/AHA guideline classes improved the specificity of cardiovascular disease event prediction (coronary artery disease, stroke, and cardiovascular death) without limiting the sensitivity. In many patients, CAC serves as a valuable tool to sharpen risk stratification.

In other patients, biomarkers may be of use to help predict the risk of ASCVD. Saeed et al. utilized the standard PCE variables in addition to N-terminal pro-hormone B-type natriuretic peptide (NT-proBNP), high-sensitivity cardiac troponin T (hs-cTnT), and high-sensitivity C-reactive protein (hs-CRP) to predict CAD, stroke, and heart failure events in older adults over a period of four years. The authors postulated that this shorter window of time, as compared to the traditional 10-year risk prediction, may be of greater value for this age group, and they found that risk prediction was improved through the addition of biomarkers, perhaps through the identification of subclinical injuries [75]. Further study is needed, but biomarker measurement may offer another way to assess older adults for potential statin benefit.

Apart from the anatomic measures of subclinical atherosclerotic disease, there are many other risk-enhancing factors that can further inform a treatment decision relating to whether to start statin therapy or otherwise intensify ASCVD risk-reduction efforts. Such factors may be lipoprotein-specific (e.g., presence of hypertriglyceridemia or elevated lipoprotein(a)), related to metabolic syndrome or renal disease, associated with inflammatory disorders (e.g., presence of rheumatoid arthritis, psoriasis, HIV, etc., or evidence of elevated high-sensitivity C-reactive protein), or nonmodifiable (e.g., South Asian ancestry, family history) [3].

While hypertriglyceridemia is not viewed to be as linearly related to ASCVD risk as LDL-C, recent guidance has confirmed that the persistence of at least moderate (150–499 mg/dL) fasting hypertriglyceridemia is an important modifiable risk factor worthy of consideration for genetic predisposition (e.g., LPL [the product of which is lipoprotein lipase], APOC3, or ANGPTL3 variants [76]), secondary causes (e.g., uncontrolled DM, certain drugs), intensive therapeutic lifestyle changes, and, if necessary, pharmacologic treatment [77]. In an analysis of coronary heart disease (CHD) risk association with hypertriglyceridemia among diverse populations, the corrected excess CHD risk conferred among adults included in the EPIC-Norfolk study (aged 65.4 ± 7.8 years) was attenuated and demonstrated lesser statistical confidence (OR 1.57, 95% CI 1.10–2.24) when compared with the effect seen (OR 1.76, 95% CI 1.39–2.21) in the somewhat younger Reykjavik study population (aged 55.8 ± 9.3 years), possibly suggesting that the hypertriglyceridemia association with excess CHD risk could be more complex and multifactorial among older adults [78]. While moderate hypertriglyceridemia is generally viewed as a risk-enhancing factor with implications for ASCVD risk reduction, when fasting triglycerides exceed 500 mg/dl, then targeted pharmacotherapy may also be considered for the purpose of attenuating the risk of hypertriglyceridemia-induced pancreatitis [77]. It is noteworthy that the presence of hypertriglyceridemia can depress estimates of LDL-C (particularly when the Friedewald equation is used), as well as the LDL-C-associated ASCVD risk, as non-LDL triglyceride-rich lipoproteins such as VLDL and chylomicron remnants also transport atherogenic cholesterol content. For those with hypertriglyceridemia, non-HDL-C may better quantitate atherogenic cholesterol, with evidence to suggest that non-HDL-C is a better predictor of ASCVD risk when the elevated vs. non-elevated status is discordant with that of LDL-C [79,80]. The treatment of hypertriglyceridemia was previously discussed in Section 3.6, with particular reiteration that omega-3 fatty acid preparations are associated with unchanged or lower, as opposed to higher, rates of bleeding in older adults [81], which may be of great relevance among those who also receive treatment with antithrombotic medications.

Lipoprotein(a) is a unique lipoprotein comprised of an LDL-like particle, the apolipoprotein B aspect of which is covalently bound via a disulfide bridge to apolipoprotein(a) (a plasminogen-like moiety), and remarkably, plasma levels of lipoprotein(a) may vary up to 1000-fold in the population [82]. Elevated lipoprotein(a) has been associated during observational and genetic studies with an increased risk for both ASCVD and valvular aortic stenosis [83], and the use of lipoprotein(a) testing in clinical practice as an ASCVD risk enhancer is growing. Aortic stenosis (AS) progression is an issue of particular relevance to older individuals, given that AS affects nearly 3% of the population aged >75 years [84] and confers a grave prognosis once symptomatic and/or associated with left ventricular dysfunction, in the absence of treatment. Lipoprotein(a) is believed to have prothrombotic properties, and lipoprotein(a) levels have demonstrated an association with AS progression after adjustment for age and cholesterol levels [85]. While statin and lifestyle therapies have little impact on lipoprotein(a) levels, which are largely genetically determined, monoclonal antibody PCSK9 inhibitors do reduce lipoprotein(a) levels, albeit with yet-unproven lipoprotein(a)-specific impacts on ASCVD outcomes [83]. Several lipoprotein(a)-targeted antisense oligonucleotides including ISIS-APO(a)_Rx_, IONIS-APO(a)_Rx_, and pelacarsen have been developed [82]. Randomized trial evidence supporting an association between targeted pharmacologic lipoprotein(a) reduction and improved ASCVD and/or AS outcomes would be confirmatory of the pathogenic nature of lipoprotein(a) [82], and careful attention to the implications for treatment among older adults is essential as this field rapidly progresses.

## 8. Concerns and Special Considerations for ASCVD Risk Reduction in Older Adults

Many individuals may experience an evolution in their values of certain risks and tradeoffs of ASCVD risk-reducing therapies during the process of aging. For example, some (particularly older) adults may place more emphasis on the small chance that statin therapy could exacerbate cognitive impairment over their desire to avoid an MI, which if suddenly fatal could even be viewed as a mechanism of death associated with minimal morbidity. Thus, the question of whether statins contribute to neurocognitive outcomes is a critical issue. To address this, a post hoc observational study of the Aspirin in Reducing Events in the Elderly (ASPREE) trial (which investigated primary prevention with aspirin) participants included 18,846 individuals aged ≥65 years who had statin use vs. nonuse at baseline [86]. Statin use was not associated with incident dementia or cognitive impairment. Furthermore, an observational study using the Korean National Health Insurance Service’s National Health Screening Cohort database suggested that statin-exposed men and women had remarkable 0.63 and 0.62 hazard ratios, respectively, for incident dementia after controlling for confounding factors [87]. In addition, a very large meta-analysis of 30 observational studies including over 9 million participants demonstrated that statin-treated patients had a lower incidence of all-cause dementia (RR 0.83) and Alzheimer’s disease (RR 0.69) [88].

Indeed, much of Alzheimer’s disease pathology has been attributed to vascular aging [89], so efforts to control atherosclerotic progression may be particularly important in individuals at risk for Alzheimer’s disease, as well as the more classical form of vascular dementia. Interestingly, some data suggest that a potential neurocognitively protective effect of statins may not necessarily be LDL-C dependent [90], although it is possibly statin intensity-dependent [91,92], rather implicating the pleiotropic and/or anti-inflammatory properties of statins. While most guidelines do not yet specifically endorse statin use for dementia prevention [93,94], these concepts should be kept at the forefront of risk-benefit discussions between clinicians and older adults contemplating alterations in statin therapy. Importantly, the ongoing Pragmatic Evaluation of Events And Benefits of Lipid-lowering in Older Adults (PREVENTABLE) trial involving the randomization of community-dwelling older (≥75 years) adults to atorvastatin 40 mg vs. placebo will specifically assess the primary endpoints of new dementia and chronic disability, which will offer high-quality evidence to help guide obligately individualized and nuanced statin therapeutic decisions in older individuals [95].

## 9. Summary

While heart-healthy lifestyle habits and statin therapy remain the mainstays of primary and secondary atherosclerotic disease prevention, pharmacotherapeutic options for lipid lowering now include a variety of effective non-statin agents such as ezetimibe, bempedoic acid, monoclonal antibody and siRNA PCSK9 inhibitors, and icosapent ethyl. Decisions regarding lipid-lowering treatments among older individuals deserve particular attention to anticipated lifespan, comorbid conditions, physical and cognitive function, independence, polypharmacy, and personal preferences informing risk-benefit tradeoffs. Historical practice patterns favoring de-intensification or the cessation of lipid-lowering therapies in older individuals may be well-intentioned, due to provider and/or patient perceptions of potential adverse drug effects such as musculoskeletal pain or cognitive dysfunction. However, a growing body of evidence suggests that the elderly population in particular may benefit from the continued pharmacologic pursuit of ASCVD prevention.

## Data Availability

Not applicable.
